# Comparative Analysis of Vehicles for the Regeneration of Mouse Endometrial Damage Model

**DOI:** 10.1007/s13770-025-00761-6

**Published:** 2025-09-06

**Authors:** Ji Yeon Han, Yoon Young Kim, Bo Bin Choi, Sung Woo Kim, Seung-Yup Ku

**Affiliations:** 1 Department of Obstetrics and Gynecology, Seoul National University Hospital, Seoul, Korea; 2 Department of Obstetrics and Gynecology, Seoul National University College of Medicine, Seoul, 03080 Korea; 3 Institute of Reproductive Medicine and Population, Medical Research Center, Seoul National University, Seoul, Korea

**Keywords:** Endometrial regeneration, Stem cell therapy, Intrauterine adhesion, Infertility

## Abstract

**Background:**

Endometrial damage is a critical factor contributing to infertility, particularly in women with refractory thin endometrium or intrauterine adhesions. Therefore, developing innovative therapeutic strategies for endometrial regeneration is essential. This study evaluates the regenerative potential of endometrial stromal cell (EMSC) injection and EMSC-loaded patch application in a mouse model with ethanol-induced endometrial damage.

**Methods:**

A mouse model of endometrial damage was established using ethanol injection into the uterine horn. EMSCs were isolated, cultured, and either HA-injected into the damaged endometrium or transplanted via a small intestinal submucosa (SIS)-based EMSC patch. Histological analyses were performed to assess endometrial thickness, gland regeneration, and fibrosis reduction.

**Results:**

Both EMSC injection and SIS-based EMSC patch engraftment promoted endometrial regeneration. However, the SIS-based EMSC patch group exhibited significant improvements in endometrial thickness, gland formation, and fibrosis reduction compared to the EMSC injection group.

**Conclusions:**

This study demonstrates the superior regenerative potential of an SIS-based EMSC patch over direct EMSC injection for endometrial repair. The findings suggest that scaffold-assisted cell therapy could be a promising approach for treating endometrial damage-related infertility. Further studies are required to optimize this strategy for clinical applications.

## Introduction

As the average age of women who marry and conceive continues to rise in some developed countries, infertility has emerged as a pressing social concern. *In vitro* fertilization (IVF) technology has advanced significantly since its inception in 1978, yielding notable improvements in outcomes. However, several challenges in IVF remain unresolved.

The endometrium, the inner epithelial layer of the uterus, consists of a basal layer and a functional layer. The basal layer contains endometrial stem cells essential for regenerating the functional layer. Endometrial decidualization is a critical process for the implantation of fertilized embryos and is fundamental to achieving a successful pregnancy [1]. Damage to the basal layer compromises the regenerative capacity of the endometrium, often resulting in conditions such as refractory thin endometrium or intrauterine adhesions, which contribute to recurrent implantation failure.

Refractory thin endometrium, often defined as an endometrial thickness below 7 mm, is widely recognized as a significant risk factor for poor IVF outcomes, though its definition remains ambiguous. Various strategies, including the use of plasma-rich plasma (PRP), low-dose aspirin, and vaginal sildenafil, have been explored to improve IVF outcomes in women with thin endometrium; however, their effectiveness in enhancing clinical pregnancy or live birth rates remains controversial and warrants further investigation [2–4]. Similarly, Asherman’s syndrome, or intrauterine adhesions, characterized by fibrous tissue bands forming within the endometrial cavity often due to uterine procedures, presents significant challenges [5, 6]. Conventional treatments, such as hysteroscopy-guided adhesiolysis with or without postoperative hormone therapy, lack consensus on optimal hormone regimens and show limited conception rates of 30%–33.3% in moderate to severe cases, with no success in recurrent cases [7–9]. Furthermore, the recurrence rate of severe adhesions is as high as 41.7% [10]. While uterine stents like hormone-releasing devices, Foley catheters, and intrauterine balloons have been explored to prevent recurrence, their efficacy remains controversial [11, 12], underscoring the need for novel therapeutic approaches to address these challenges.

Recent advancements in experimental models have significantly enhanced the field of endometrial regeneration, particularly through research utilizing rodent models. Therapeutic strategies now encompass a wide array of techniques, including stem cell therapies, growth factor administration, and innovative approaches such as tissue engineering, bioengineering solutions, organoids, and PRP application [13]. Notably, in rat models of endometrial regeneration, synergistic effects have been observed with the combined use of collagen scaffolds, growth factors, and stem cells, yielding promising results [14–17]. These studies predominantly utilized mechanically induced endometrial damage. However, ethanol-induced endometrial damage, as demonstrated by Kim et al. [18], has emerged as a potentially superior model, offering advantages over mechanical methods. Thus, validating the aforementioned therapeutic approaches in alternative endometrial damage models is critical to ensure their efficacy and safety.

Hyaluronic acid (HA) is a natural glycosaminoglycan known for its high biocompatibility, water retention capacity, and ability to mimic the extracellular matrix (ECM), making it suitable for supporting cell survival and migration [19]. Small intestinal submucosa (SIS), derived from porcine tissue, is a collagen-rich ECM scaffold that not only provides mechanical support but also contains bioactive molecules such as growth factors, which can promote angiogenesis and tissue remodeling [20]. These properties make HA and SIS ideal candidates for evaluating regenerative approaches in the damaged endometrium.

The objective of this research is to evaluate the therapeutic potential of endometrial stromal cell (EMSC) injection, either alone or in combination with SIS-based patch application, in mouse models of ethanol-induced endometrial damage. To precisely control the extent of uterine injury, surgical techniques are employed to induce endometrial damage, thereby minimizing confounding variables associated with the method of damage induction.

## Materials and methods

### Production of the uterine damage model

The mouse uterine damage model was produced using the previously described protocol. Briefly, 6-week-old female C57BL/6 mice were anesthetized with isoflurane and maintained. Then, the uterine horn was exposed with a dorsal approach, and 0.1 ml of 50% ethanol (EtOH, Sigma-Aldrich, St. Louis, MI, USA) was injected using a 26G 1/2 inch needle [18]. The EtOH was incubated for 10 min, and the approached region was closed and stapled with a clip. The mice were recovered in an isolated cage. The IACUC [19-0173-S1A0(2)] of Seoul National University Hospital approved all the animal experiments.

### Isolation and culture of endometrial stromal cells (EMSCs)

The uterine horn of the 6-week-old female C57BL/6 was removed and washed using HBSS (Invitrogen, Waltham, MA, USA). The lipid pad was moved and cut into pieces using a surgical blade. Pieces were digested with collagenase I (1 mg/ml, Invitrogen) for 2 h at 37 °C. After 2 h of incubation, repeated pipetting reconstituted the digest into small pieces. The digest was filtered through a 40-um cell strainer and centrifuged for 5 min at 3000 rpm, room temperature. The collected pellets were reconstituted in EMSC culture media, constituted with DMEM/F12 media (Invitrogen) supplemented with 10% fetal bovine serum (FBS; Invitrogen) and 100 mM of Penicillin/streptomycin (Invitrogen). The cells were replated, and the media was exchanged every other day.

### Preparation of small intestinal submucosa (SIS)-based EMSC patch

Small Intestinal Submucosa (SIS) was purchased from Cook Medical Company (Bloomington, IN, USA) and prepared to the proper size using a biopsy punch 3.0 mm. Pieces of SIS were put into each well of 4-well dishes and pre-soaked with EMSC culture media.

### Preparation of hyaluronic acid (HA) scaffolds—EMSC gel

Cultured cells were dissociated, collected by centrifugation, and mixed with media and/or hydrogel. The injectable hydrogel was purchased from Il Dong Pharmaceutical (HyFence, Il-Dong Pharmaceutical Co. Ltd, Seoul, Korea) and warmed at RT before mixing with cells. Each group was prepared as a mixture of 100 μL of media at room temperature.

### SIS-based EMSC patch application and EMSC injection

**Fig. 1:**
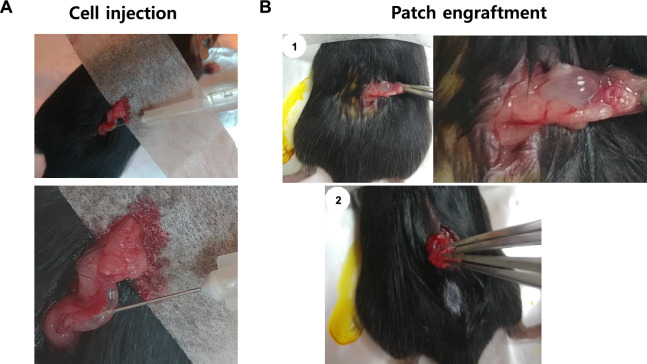
Experimental scheme of this study

The dorsal region of the mouse was opened, and either an SIS-based EMSC patch or an HA-EMSC was transplanted to the damaged region produced by previous damage induction. SIS-based EMSC patch was put into the region and attached without any stitches. For the HA-gel injection, HA-gel was loaded into a syringe with a 32-gauge needle and injected. After the engraftment, the open region was closed using a clip. An overall schematic of the experimental design is provided in Fig. 1.

### Histological analyses of uterine tissue

Damage and regeneration of the uterine endometrium were analyzed using Haematoxylin and Eosin (H&E) staining and Mason Trichrome (MT) assay. Briefly, isolated tissue samples were fixed with 4% paraformaldehyde (PFA; Sigma-Aldrich) for 24 h and washed with PBS. Then, samples were put into a paraffin block and processed for further H&E staining or immunohistochemistry.

### Statistics

Statistical analysis was performed using analysis of variance (ANOVA), followed by Bonferroni’s test for multiple comparisons, utilizing GraphPad Prism 9.0 (GraphPad Software, San Diego, CA, USA). A *p*-value of less than 0.05 was considered statistically significant.

## Results

### Treatment of damaged model by EMSC injection or SIS-based EMSC patch engraftment

**Fig. 2:**
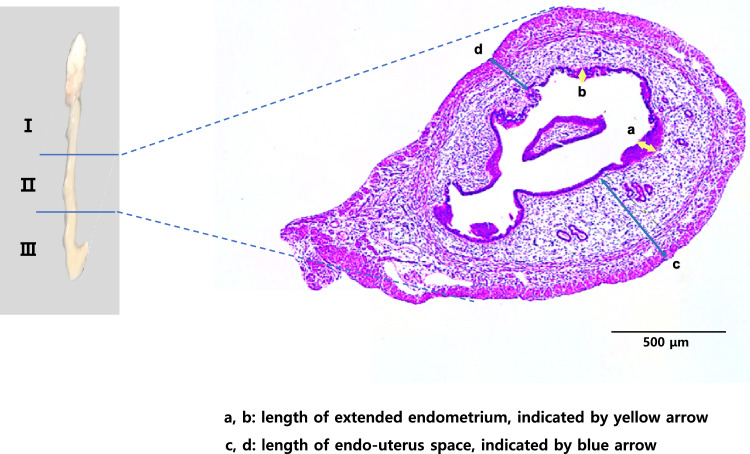
Procedures of EMSC injection and SIS-based EMSC patch engraftment in the murine model. The uterine horn was exposed by dorsal approach and treated either by EMSC injection or SIS-based EMSC patch engraftment. **A** EMSC injection for damaged uterine horn, **B** SIS-based EMSC patch engrafted to damaged uterine horn

Our group previously reported on the generation of uterine damage mouse models. Damaged models were prepared by the dorsal approach during the operation. After 2 weeks of damage induction, HA-embedded EMSC (*n* = 5, Fig. 2A) or an SIS-based EMSC patch (*n* = 5, Fig. 2B) was transplanted in the region of damage-induced endometrium. The regeneration of the endometrium was further analyzed.

### Measurement of endometrial thickness and glands

**Fig. 3:**
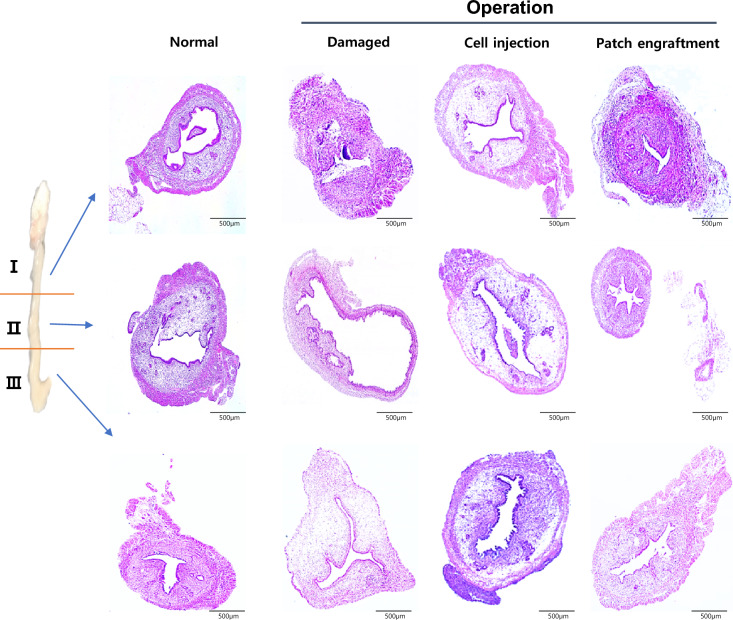
Measurement of the endometrial thickness and endometrium-myometrium interface. The region measured as the thickness of the extended endometrium (yellow arrow) and the endometrium-myometrium interface (blue arrow) is indicated, and the arrow indicates the diameter evaluated

Damage induction and EMSC injection or SIS-based EMSC patch engraftment were performed at the middle region of the uterine horn (region II, Fig. 3). The lengths of the extended endometrium (yellow arrow) and the endometrium-myometrium interface (blue arrow) were measured as indicated in Fig. 3.

### Regeneration of the endometrium due to the cell engraftment method

**Fig. 4:**
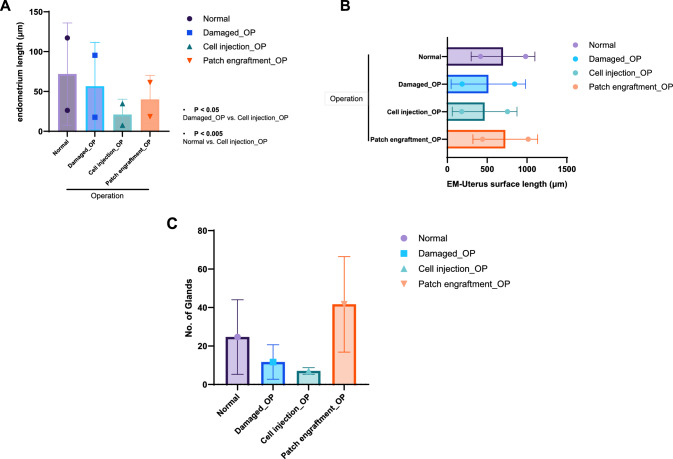
Histological analysis of the endometrium. Structural alterations and regenerations of endometrium were analyzed in treated group either by EMSC injection or SIS-based EMSC patch engraftment

The damaged and regenerated region (II) of each group was evaluated by histology (Fig. 4). Induction of endometrial damage was confirmed in the damaged group compared to the normal control. The normal uterus demonstrated a thick endometrial layer in the center of the uterine horn. In contrast, the endometrium layer in the damaged group was visibly damaged and overall thinner compared to the normal control. The damaged group also displayed disrupted endometrial architecture and significant thinning of the functional layer, further confirming successful model induction. In contrast, both treatment groups showed improved histological appearance, with the patch group exhibiting more organized glandular structures and thicker endometrial layers compared to the cell-injected group.

**Fig. 5:**
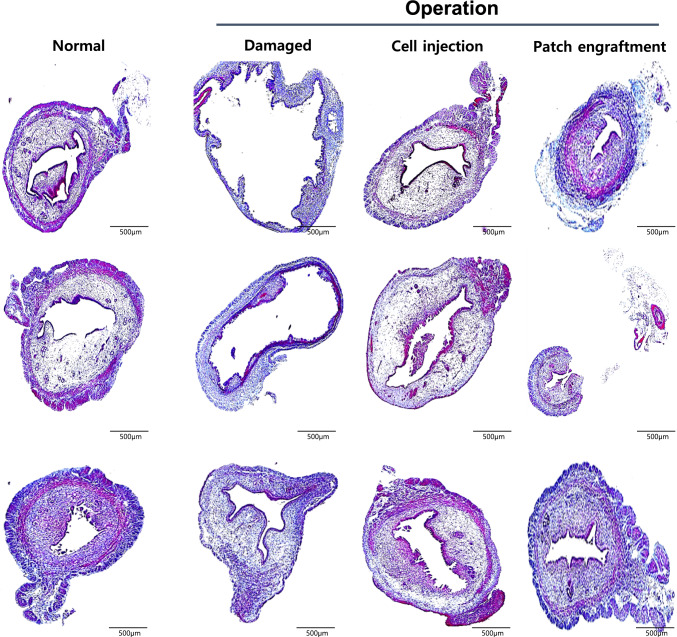
Measurement of regenerated endometrium after treatment. **A** Measurement of extended (regenerated) endometrium thickness, **B** Measurement of extended (regenerated) endometrium-myometrium interface, **C** Number of endometrial glands after EMSC injection or SIS-based EMSC patch engraftment

Some regions in the endometrium demonstrated partial regeneration, and both groups regenerated after EMSC injection or SIS-based EMSC patch engraftment. Regeneration of the EMSC injection or SIS-based EMSC patch group was further measured by the thickness of the extended endometrium and space between the surface and endometrium. The thickness of the endometrium was longer in the SIS-based EMSC patch group. However, the regeneration degree was close to normal endometrium (Fig. 5A). The thickness of the endometrium-myometrium interface increased in the SIS-based EMSC patch group, and the regeneration degree was relatively similar to that of the standard control (Fig. 5B).

The number of glands was also measured and was significantly increased in the SIS-based EMSC patch group (Fig. 5C).

### Reduction of fibrosis

Fibrosis is one of the primary reasons for the dysfunctionality of the endometrium; therefore, measuring the degree of fibrosis based on the collagen content is a significant indicator of fibrosis. The degree of the positively stained color (visualized as deep blue) correlated with the deposition of collagen fiber.

**Fig. 6:**
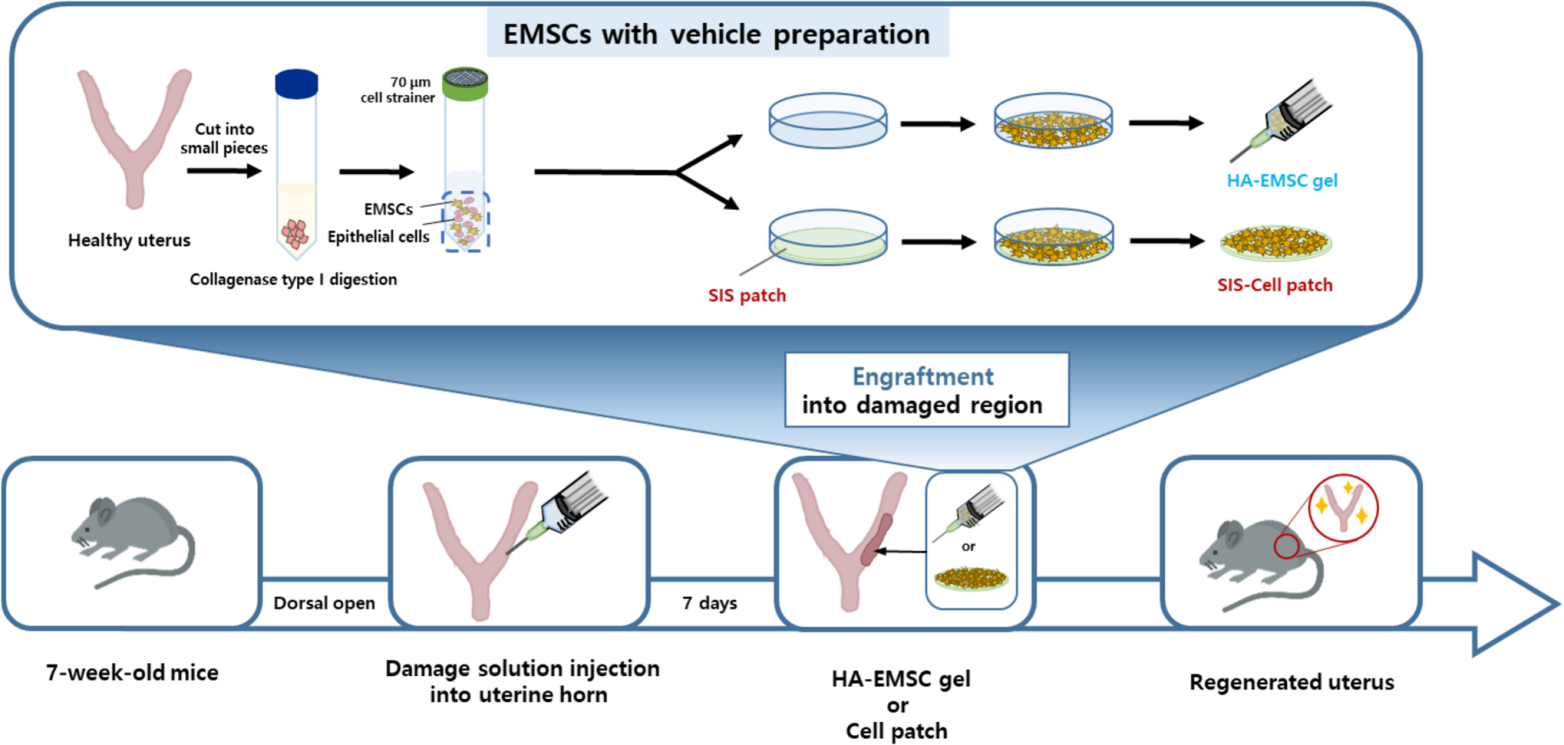
Degree of fibrosis in regenerated endometrium. Proportion of collagen in endometrium was analyzed by Mason Trichrome staining

Masson trichrome staining evaluated the proportions of collagen in each normal, damaged, and treatment group. It revealed dense collagen deposition in the damaged group, which was visibly reduced following EMSC treatment (Fig. 6). Treatments of EMSC, either by EMSC injection or SIS-based EMSC patch engraftment, reduced the collagen content. Overall, the collagen content was significantly reduced in the cell-treated group; however, the patch group demonstrated the lowest collagen density, indicating a greater degree of fibrosis resolution.

## Discussion

To the best of our knowledge, this study is the first to confirm the therapeutic efficacy of an EMSC-loaded patch utilizing SIS for the repair of endometrial damage in a murine model. Our findings demonstrate significant regenerative effects of *in vitro*-processed isotopic cells encapsulated within composite scaffold materials. The SIS-based EMSC patch more effectively promoted endometrial regeneration than EMSC injection alone. Additionally, fibrosis reduction was more pronounced, with lower collagen content in the SIS-based EMSC patch group. These findings suggest that SIS-based EMSC patch engraftment provides superior therapeutic benefits for endometrial repair.

EMSCs, derived from the endometrial stromal compartment, offer distinct advantages over other mesenchymal stem cell sources such as adipose-derived, bone marrow-derived, or umbilical cord-derived stem cells. Their inherent tissue specificity, natural cyclic regenerative capacity, and reduced immunogenicity make them particularly well-suited for endometrial repair.

Our previous study demonstrated that decidualized EMSCs encapsulated in HA hydrogel effectively promoted endometrial regeneration in a murine model [21]. This approach enhanced endometrial thickness, accelerated recovery, and improved fertility outcomes. One notable distinction from the current study is the choice of scaffold—our previous research used HA hydrogel, while this study employs a patch-based system with SIS.

Studies have shown that SIS promotes endometrial regeneration, prevents intrauterine adhesions, increases endometrial thickness, and improves receptivity by enhancing the expression of uteroglobin and HOXA10 [22]. Additionally, SIS has been explored in combination therapies, such as with intrauterine balloons for fibrosis repair [23] and as a scaffold for seeding stem cells like umbilical cord mesenchymal stem cells (UC-MSCs) to support uterine tissue engineering [24]. In addition to its mechanical advantages, SIS contains a variety of bioactive molecules, including vascular endothelial growth factor (VEGF), fibroblast growth factor-2 (FGF-2), transforming growth factor-beta (TGF-β), and epidermal growth factor (EGF) [20]. These bioactive molecules may contribute to the superior therapeutic efficacy of the SIS-based EMSC patch group by enhancing angiogenesis, tissue regeneration, and cell-mediated remodeling beyond mere structural support.

Both HA hydrogels and SIS share biocompatibility, support for cell growth, and applications in tissue engineering. However, they differ in composition, mechanical properties, degradation rate, and cell interaction. HA hydrogels, made of polysaccharides, are soft, degrade quickly, and may require modification for cell adhesion. In contrast, SIS, composed of collagen-rich extracellular matrix, provides greater mechanical support and naturally promotes cell attachment. Our previous study utilized HA hydrogels as a scaffold for EMSCs due to their ability to mimic the extracellular matrix. However, their rapid degradation and lower mechanical strength limited long-term structural support. In this study, SIS was selected for its durability and enhanced support, enabling prolonged cell engraftment and more effective endometrial regeneration in the mouse model.

In addition to their structural compatibility with these biomaterials, EMSCs may exert therapeutic effects through paracrine signaling, including secretion of pro-angiogenic factors, suppression of fibrosis, and recruitment of endogenous progenitor cells.

Several studies highlighted the regenerative potential of stem cell-loaded cell patches for treating damaged endometrium [25]. Collagen patches loaded with adipose-derived mesenchymal stem cells (ADSCs), menstrual blood-derived stem cells (MenSCs), bone marrow mesenchymal stem cells (BMSCs), and human embryonic stem cell (hESC)-derived endometrium-like cells were shown to significantly enhance endometrial regeneration in rat models [14, 16, 26, 27]. However, these studies are limited by their reliance on heterotopic cells. In contrast, the present study employed bioprocessed isotopic cells for the treatment of damaged uteri, demonstrating effective regenerative therapeutic outcomes and direct relevance to the endometrium.

Previous studies utilizing isotopic cells for the treatment of damaged endometrium have several limitations. While direct cell injection methods, such as those employed by Bausyte et al. [28], demonstrated the regenerative potential of human endometrium-derived mesenchymal stromal cells (hEnMSCs), they lacked a structured scaffold, which may have led to lower cell retention and limited long-term therapeutic effects. In contrast, this study introduced a patch-based approach using SIS as a scaffold, providing a more supportive microenvironment for EMSCs and enhancing regeneration efficiency.

Furthermore, the chemically induced ethanol injury model used in this study offers greater reproducibility and consistency compared to mechanical injury or chemotherapy-based models, which can introduce variability in tissue damage and recovery [18, 29]. By employing a controlled injury method and a structured delivery system, this study provides robust evidence supporting the use of SIS-based EMSC patches as a more effective strategy for endometrial regeneration. These findings emphasize the therapeutic potential of scaffold-assisted cell engraftment in overcoming the limitations of traditional stem cell-based treatments for endometrial repair.

In clinical applications, Tersoglio et al. demonstrated the regenerative potential of endometrial mesenchymal stem cells (enMSCs) injections combined with platelet-rich plasma (PRP) in women with recurrent implantation failure [30]. However, this approach raises the possibility that PRP, rather than enMSCs alone, played a significant role in endometrial regeneration. PRP contains growth factors and cytokines that stimulate angiogenesis, tissue repair, and inflammation reduction [31], making it challenging to isolate the specific regenerative effects of enMSCs. In contrast, this study employed a structured SIS scaffold without PRP, demonstrating enhanced cell retention, fibrosis reduction, and gland regeneration, purely through EMSC-SIS interactions.

A cautious interpretation of the results is necessary due to several key limitations. First, pregnancy outcomes were not assessed, making it unclear whether endometrial regeneration translated into improved fertility. Follow-up research will evaluate the long-term effects of SIS-based EMSC patch engraftment on reproductive outcomes. Second, the study involved two surgical procedures, one for endometrial damage induction and another for SIS-based EMSC patch engraftment, which may have introduced additional uterine injury. Moreover, the procedural differences between the EMSC injection group and the SIS-based EMSC patch group represent a potential limitation of the study. However, despite this, the SIS-based EMSC patch group exhibited significant regeneration, suggesting that the therapeutic effects of the SIS-based EMSC patch may have been underestimated. If this procedural factor is corrected, the actual efficacy of the SIS-based EMSC patch could be even greater. Third, direct clinical translation is limited, as the SIS-based EMSC patch must be optimized for human single-uterus anatomy before application. Further research is required to refine the patch size, structure, and implantation method to ensure effective adaptation for human treatment.

These findings highlight the potential of SIS-based EMSC patches as an advanced regenerative therapy for endometrial repair. By addressing the limitations of traditional EMSC injection methods, this approach enhances cell retention, reduces fibrosis, and promotes gland regeneration, offering a promising foundation for future translational research. Further investigations will focus on optimizing clinical application strategies, evaluating pregnancy outcomes, and refining scaffold design to ensure seamless adaptation for human use. Ultimately, this study provides crucial insights into scaffold-assisted cell patch therapies, paving the way for more effective treatments for endometrial dysfunction and infertility.

## Data Availability

The data supporting this study's findings are available from the corresponding author upon reasonable request.
